# An Unusual Presentation of Kikuchi-Fujimoto Disease with Recurrent Subdural Effusion

**DOI:** 10.7759/cureus.2302

**Published:** 2018-03-10

**Authors:** Sara Shahid, Syed H Alam, Indira Hadley

**Affiliations:** 1 Medicine, Lahore Medical And Dental College, Lahore, Pakistan; 2 Department of Rheumatology, Temple University Hospital; 3 Department of Rheumatology, John H Stroger J. Hospital of Cook County

**Keywords:** kikuchi-fujimoto, subdural effusion, lymphadenopathy, histiocytic necrotizing lymphadenitis

## Abstract

A 24-year-old man complained of a right temporal headache for four weeks. The patient denied any trauma or previous anticoagulation use. He also reported tender right facial swelling. His physical exam was unrevealing except for right cranial nerve (CN) VI palsy, right parotid enlargement, and cervical adenopathy. Laboratory findings were significant for mild leukopenia at 3300 cells/uL. The computed tomography (CT) scan obtained showed a chronic left subdural effusion with a 4 mm midline shift and confirmed right parotid enlargement and cervical lymphadenopathy. Surgical burr hole evacuation was done and the fluid was sent for wound culture analysis. The infectious diseases service recommended initiating antibiotics, which were later stopped due to cerebrospinal fluid (CSF) cultures with no growth of any organisms. His CN VI palsy resolved during admission. The patient was discharged with follow-up for biopsy. The patient was lost to follow-up. The patient presented to the emergency department (ED) three months later, with a left-sided frontal headache. A repeat CT scan showed a new, right-sided fluid collection outside the brain parenchyma. Burr hole evacuation was done again and purulent fluid was drained. Antibiotics were held this time, but anti-tuberculous therapy was initiated empirically. The otolaryngology service was consulted and a lymph node biopsy was performed. The pathology showed histiocytic necrotizing lymphadenitis. A dural biopsy was done as well and was consistent with histiocytic necrotizing lymphadenitis involving the dura. Cultures from the subdural fluid did not grow any organism. The patient remained neurologically intact. He improved after surgery was done to drain the fluid and was managed by analgesics. The cultures from the extra-parenchymal fluid collection remained negative for pathogens and tuberculous mycobacteria. The patient was discharged with rheumatology clinic follow-up. He saw the rheumatologist six weeks after the discharge. During his clinic visit, the patient reported no recurrence of headaches, fevers, rash, or joint pain. Our patient had a rare presentation of Kikuchi-Fujimoto disease, in which he had a subdural fluid collection resulting in neurological complications that required surgical intervention.

## Introduction

Kikuchi syndrome, also known as Kikuchi-Fujimoto disease, was first described in the literature by Kikuchi and Fujimoto et al. It is a rare disorder, and, histopathologically, it is a histiocytic necrotizing lymphadenitis. In this disease, the most common presentation is lymphadenopathy, and the patient may also be having accompanying constitutional symptoms in varying frequency, such as fever, weight loss, and night sweats [1]. Kikuchi’s syndrome has been described to have an association with the development of systemic lupus erythematosus (SLE) [1]. The involvement of the central nervous system (CNS) has also been reported and can be a harbinger of significant morbidity and even mortality, in a disease whose course is otherwise benign in most cases [2]. Hence, it needs to be addressed promptly. This presentation has been previously described in three case reports, two of them being adult patients [3-4] and one was a pediatric patient [1]. In these case reports, the patients presented with subdural effusions and aseptic meningitis, and an analysis of the latter typically shows lymphocytic pleocytosis and slightly elevated glucose and protein levels [1,3-4]. Our case is unique, as the patient had a recurrent involvement of the CNS and the subdural effusion recurred on the side opposite to where it first presented. To our knowledge, this presentation has not been described in the literature earlier.

## Case presentation

History

A 24-year-old Filipino man with no significant past medical history began complaining of a constant, 7/10 intensity, right temporal headache to his primary physician about two weeks prior to visiting him. A computerized axial tomography scan was obtained, which showed a chronic subdural fluid collection in the left frontal, parietal, and temporal lobes, with a 4 mm midline shift to the right, along with right parotid enlargement and cervical lymphadenopathy. On a review of symptoms, the patient denied any trauma, falls, fights, previous anticoagulation use, and drug abuse. He also denied any numbness, motor weakness, syncope, rash, or bladder incontinence. He endorsed an intermittent blurring of vision and nausea. He also reported tender right facial swelling for two months and subjective fevers.

Examination

On a physical exam, the patient was found to be afebrile. The rest of his vital signs were normal. The examination was also significant for papilledema and right cranial nerve VI palsy, with no other neurological deficits. Laboratory findings showed sodium 140 mEq/L, potassium 3.7 mEq/L, chloride 105 mEq/L, bicarbonate 26 mEq/L, BUN 11 mg/dL, creatinine 0.7 mg/dL, white cells 3300/uL, hemoglobin 14 g/dL, mean corpuscular volume 84.7 fL, and platelets 155,000/uL.

Initial management

The patient was taken to the operating room for burr hole evacuation and 50 ml of purulent fluid was drained. The fluid was sent for wound culture, including fungal cultures. The infectious disease service was consulted, which recommended initiating the antibiotics vancomycin and meropenem, which were later stopped due to normal cerebrospinal fluid cultures, and the patient developing a macular skin rash to the antibiotics. During his hospital stay, the patient remained afebrile and neurologically intact. His CN VI palsy resolved. The patient was discharged with follow-up to the infectious disease, neurosurgery, and otolaryngology clinic for a possible biopsy of the parotid gland and cervical lymph nodes. Suspicion for tuberculosis was high, but treatment was held, as the AFB cultures in subdural fluid were negative, pending growth. The patient was lost to follow-up after hospital discharge.

Recurrence

The patient presented to the emergency department three months after his initial hospital stay with a left-sided frontal headache. He reported that his right face swelling and headaches initially improved after his hospital discharge, but the symptoms recurred one week prior to this presentation. A repeat CT scan showed new, right, frontal extra-axial fluid collection with 3 mm midline shift, with a resolution of the previous left frontoparietal fluid. Another burr hole was done by neurosurgery and purulent fluid was drained. At this point, antibiotics were held, but anti-tuberculous therapy was initiated according to infectious disease service recommendations and pending final growth of the organisms.

Pathology

Otolaryngology service was consulted during this hospital stay, and a right level V cervical lymph node biopsy was performed. The pathology of the biopsy showed histiocytic necrotizing lymphadenitis. A dural biopsy was also sent at the time of the burr hole, which showed multifocal chronic inflammation with the debris of necrotic inflammatory cells consistent with Kikuchi’s disease involving the dura. Cultures from the subdural fluid did not grow any organisms.

Further management and follow-up

During the second admission, the patient had one temperature reading of 101.8 F, which was on the first postoperative day, and apart from that, he remained afebrile. He remained neurologically intact and was managed by analgesics. The patient was discharged with rheumatology clinic follow-up. He saw the rheumatologist six weeks after the discharge. During his clinic visit, the patient reported no recurrence of headaches, fevers, rash, or joint pain. The cultures from the extra-axial fluid collection remained negative for pathogens and tuberculous mycobacteria. Based on infectious disease recommendations, anti-tuberculous therapy was stopped. The patient had additional labs drawn in the clinic, the results of which were insignificant. These included an erythrocyte sedimentation rate (ESR) of 16 mm/hr, C-reactive protein (CRP) of 0.03 mg/dL, creatine kinase (CK) of 58 U/L, and normal complements. The patient did not have any signs or symptoms of systemic lupus erythematosus (SLE) on exam or history, and he was planned to be monitored for the symptoms of SLE on following visits.

## Discussion

Kikuchi’s syndrome (histiolytic necrotizing lymphadenitis) was first described by Masahiro Kikuchi and Y. Fujimoto et al. in 1972. The disease usually affects young adults and the mean age at diagnosis is 25 years [1] with a male to female ratio of 1.4:1 [5]. Regarding the etiology, various infections have been postulated to play a role, such as toxoplasma, brucellosis, Bartonella henselae, Yersinia enterocolitica, and viruses such as herpes viruses, human immunodeficiency virus (HIV), Epstein-Barr virus, rubella, paramyxovirus, and parainfluenza virus [6]. Studies have also indicated a possible role for interferon gamma, interleukin-6, and apoptotic cell death [7-8]. In the study by Kubota et al. [7], the elevated serum levels of interferon gamma and IL-6 were observed in the acute phase of the illness, which returned to normal in the convalescent period. In the same study, interferon alpha, tumor necrosis factor, and IL-2 were within the normal range. The interferon-gamma and IL-6 levels returned to normal during convalescence. It has been hypothesized by some authors that Kikuchi syndrome may represent the body’s autoimmune response to various stimuli. Numerous inciting agents have been proposed, including the bacterial and viral agents listed above. Due to the increased volume of cases in Japan, some papers have suggested the role of certain dietary sources, such as raw fish. Kikuchi syndrome shares sex and age predisposition, along with histologic features, with systemic lupus erythematosus (SLE). The disease presents with cervical lymphadenopathy, commonly in the posterior cervical triangle and the jugular carotid chain [6]. Lymphadenopathy may be seen in parotid glands as well [5]. Extranodal involvement of the lungs, with interstitial lung disease, bone marrow, and the musculoskeletal system with rash and arthralgias may also be seen [5]. Patients may also present with non-specific symptoms of a chronic inflammatory disease, such as pyrexia and weight loss [5]. No definite lab test is a hallmark of Kikuchi’s syndrome, but patient findings include leukopenia, which is found in 25.5% to 58.3% of patients [2]. Confirmatory diagnosis is done by lymph node biopsy, which shows a distortion of the normal nodal architecture with cortical and paracortical nodules. The nodules are necrotic with karyorrhectic debris from apoptosis. The foci of necrosis can be isolated or include larger areas. Other features include proliferated histiocytes, immunoblasts, T cells expressing CD8+, and the absence of eosinophils or neutrophils [5].

Despite the large number of histopathological features described in the literature, the minimum criteria for the diagnosis of Kikuchi’s syndrome are the presence of aggregated histiocytes with occasional crescent-shaped nuclei, plasmacytoid histiocytes, and scattered karyorrhexis [5]. It has been recommended that the differential diagnosis of Kikuchi’s syndrome should include malignant lymphoma, metastatic carcinoma, SLE, toxoplasmosis and cat-scratch disease, and acquired immunodeficiency syndrome [5,9-10].

Kikuchi’s syndrome has a good prognosis in which the lymphadenopathy is usually self-limited and almost always resolves in six months [1]. However, there have been case reports of Kikuchi’s syndrome recurring up to eight years after the initial presentation. The treatment for Kikuchi’s syndrome is essentially observation and symptomatic control with analgesics and anti-inflammatory medications [5]. However, in cases of complicated Kikuchi’s syndrome, case reports have demonstrated the use of glucocorticoids or hydroxychloroquine, with considerable success [9]. Yoshioka et al. [9] treated 13 patients with Kikuchi disease and prolonged fever with methylprednisolone for three days at a dose of 0.5 grams/day. In this case, a resolution of fever was seen in all patients, and four out the 13 patients had a relapse of fever. Rezai et al. [10] treated a patient with Kikuchi disease who had systemic symptoms with chloroquine for four days and rapid response was seen. There was a recurrence, which was then treated with 14 days of hydroxychloroquine at a dose of 200 mg twice a day. In this case, the patient’s symptoms resolved in 12 hours with hydroxychloroquine and on follow-up until 14 months, they did not recur.

There have been cases reporting success with the use of intravenous immunoglobulin and minocycline. To our knowledge, there have been three cases of Kikuchi’s syndrome presenting with neurological complications [1-3]. Of the cases, two were adult patients [3-4] and one was a pediatric patient aged 13 [1]. The neurological complications in all three cases were subdural effusions, with either subdural empyema or hematoma. In our patient, his dural biopsy showed fibrous tissue with multifocal chronic inflammation, which is consistent with other cases in which the dura was involved due to Kikuchi’s syndrome. This is similar to what was seen in the case of Santos et al. [1]. Our patient had a rare presentation of Kikuchi’s, in which he had a subdural fluid collection resulting in neurological complications that required surgical intervention. It was also very unusual that he had a recurrence of the subdural effusion on the opposite side. This second presentation of a contralateral subdural hematoma with recurring parotid swelling represents either an incomplete resolution of the disease or recurrence, in which the latter has been described in less than 10% of patient’s with Kikuchi’s syndrome. Thus far, the patient has remained symptom-free after his second episode of Kikuchi’s syndrome and will continue to follow in the rheumatology clinic for regular check-up and monitoring. Due to its association with SLE [1], the patient will be monitored for symptoms of SLE on future visits.

## Conclusions

We describe a case of Kikuchi-Fujimoto disease, presenting as parotid gland swelling, lymphadenopathy, and subdural effusion. The diagnosis was based on characteristic histopathology, which is histiocytic necrotizing lymphadenitis. Our case was unique due to the involvement of the central nervous system with Kikuchi-Fujimoto disease. This has been described three times in literature, prior to our case. Our patient's biopsy from the dura was consistent with dural involvement by histiocytic necrotizing lymphadenitis. The patient improved after a surgical evacuation of subdural effusion and conservative management. Further observations are needed to better define the course of this rare disease and to optimize its management. Studies are also needed to elucidate potential associations with other autoimmune diseases.
